# Addressing the Quality of Hospital Care of Colorectal Cancer Patients Undergoing Surgery: What Can We Learn From the National Bowel Cancer Audit?

**DOI:** 10.7759/cureus.22333

**Published:** 2022-02-17

**Authors:** Chunhei Li, Setthasorn Zhi Yang Ooi, Timothy Woo, Hei Man Priscilla Chan

**Affiliations:** 1 Department of Surgery and Cancer, St. Mary's Hospital, London, GBR; 2 School of Medicine, Cardiff University, Cardiff, GBR; 3 Department of General Surgery, Prince Charles Hospital, Merthyr Tydfil, GBR

**Keywords:** quality outcomes, general surgery, hospital care, colorectal cancer, national bowel cancer audit

## Abstract

Introduction

The National Bowel Cancer Audit (NBOCA) is the largest database in the United Kingdom that audits real-world data and allows comparison of the quality of care for colorectal cancer patients. This study aimed to highlight relevant clinical factors in the NBOCA that contribute to variation in the quality of care provided in different hospitals.

Methods

Data from 36,116 patients with colorectal cancer who had undergone surgery were obtained from the NBOCA. These were patients from 145 and 146 hospitals from the years 2016 and 2017, respectively. A validated multiple linear regression was performed to compare the identified clinical factors with various quality outcomes. The quality outcomes defined in this study were the length of hospital stay of more than five days, two-year mortality, 30-day unplanned readmission rate, 90-day mortality, and 18-month stoma rate.

Results

Four clinical factors (laparoscopy rate, abdominal-perineal-resection-of-rectum, pre-operative radiotherapy, and patients with distant metastases) were shown to have a significant (p < 0.05) impact on the length of hospital stay of more than five days and the 18-month stoma rate. The 18-month stoma rate was also a significant predictor (p < 0.001) with two-year mortality.

Conclusion

The NBOCA should consider adjusting for these factors when reporting the quality of care provided in hospitals. Hospitals should monitor the four clinical factors for colorectal cancer patients during perioperative care. When formulating a management plan for patients with colorectal cancer, clinicians should consider these factors along with the individual patient's history.

## Introduction

The National Bowel Cancer Audit (NBOCA) project is one of the largest national annual audits that records open data for patients diagnosed with colorectal cancer in the United Kingdom (England and Wales) to facilitate the improvement of care [1]. Since 2004, it has annually recorded 300,000 to 450,000 colorectal cancer cases from over 45 participating trusts. The NBOCA addresses the quality of care provided in hospitals by measuring consensual parameters that reflect the patients’ oncological and surgical care and quality of life. This provides a benchmark of the clinical performance in individual trusts, which supersedes the isolated performance appraisal on colorectal surgery practice [1,2]. The NBOCA records a diverse range of parameters that are related to the quality of clinical care for cancer patients. To facilitate the interpretation of such extensive information, the NBOCA publishes a report annually on their website that summarises the key findings. Additionally, the NBOCA has also created an online search engine that enables individual hospitals to be searched and compare their standards against the national standard for that particular year.

An important indicator measured in the NBOCA that represents the overall oncological care for patients with colorectal cancer is the long-term survival rate, which is usually reflected by two-year mortality. Other markers include 90-day mortality and a 30-day unplanned readmission rate, both of which provide a snapshot of the quality of the operation and its complication profile [1,3]. In addition to mortality, the morbidity outcome is also essential to determining the quality of life after an operation. This includes the permanent stoma rate recorded at 18 months and the hospitalisation duration of more than five days [1,3]. Collectively, all these parameters are perceived as quality outcomes of the care provided by hospitals [1].

However, the diverse range in the reported quality of care among different hospitals raises a topic for discussion. In the recent NBOCA report, even after adjustment, 90-day mortality ranged from 0% to 11%, 18-month stoma rate ranged from 25% to 89%, 30-day unplanned admission rate ranged from 0% to 25% variation and two-year mortality ranged from 5% to 43% [4]. These figures have prompted the need to define and explore the underlying core clinical factors that might contribute to these varying results.

Of note, even though the NBOCA currently adjusts these performance standards of hospitals according to patient volume and the annual number of operations performed at the hospital, this only accounts for the difference between high-volume and low-volume surgical centres. This may not be sufficient to address the variation in percentages observed. Therefore, this study aims to identify the most relevant clinical factors for the overall quality outcomes for colorectal cancer patients undergoing surgeries in different hospitals. It is hypothesised that these clinical factors should also be adjusted for in the NBOCA’s annual report.

## Materials and methods

Data selection 

**Figure 1:**
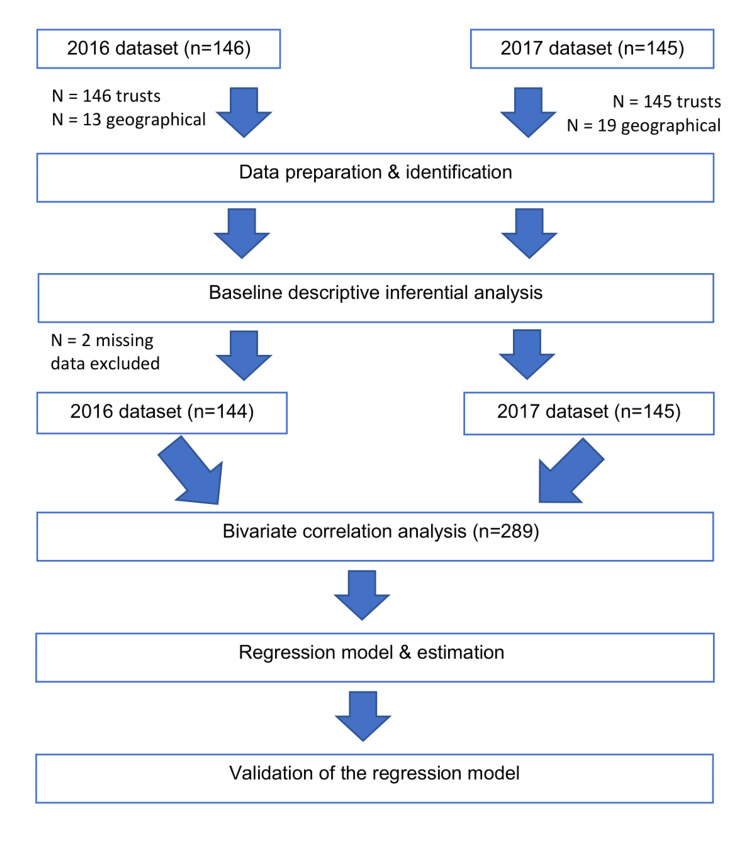
Flow diagram demonstrating the steps for the analysis.

This was a retrospective study using data obtained from the NBOCA public database for the years 2015/2016 and 2016/2017 in England, which is the most up-to-date data accessible by the public (Figure 1). All the parameters recorded in the NBOCA data sheet were included in this study and treated as clinical factors. A clinical factor is defined as a potential parameter that could affect the quality of the outcome for colorectal cancer patients. 

The five quality outcomes in the NBOCA are defined as: oncological outcomes (two-year mortality), morbidity outcomes (length of hospital stay of more than five days and an 18-month stoma rate), and surgical outcomes (30-day unplanned readmission and 90-day mortality).

Missing data and outliers 

Hospitals with missing data were addressed by pairwise or test-by-test deletion. All univariate outliers were highlighted and computed for regression analysis. 

Statistical analysis 

SPSS (IBM, NY, USA) and R program were used for data analysis and graph production. Spearman's rank correlation coefficient was used for bivariate correlation analysis. Multiple linear regression was performed to estimate the association between the significantly correlated variables. All the variables underwent testing for normality, linearity, homoscedasticity, and multicollinearity. Optimised regression models were selected. The adjusted R^2^ and beta values were calculated. The performance of the regression models was evaluated and externally validated on a randomly selected dataset from NBOCA. The root mean square error (RMSE) was calculated to validate the accuracy of the regression model. A p-value of less than 0.05 was considered statistically significant after post hoc adjustment.

## Results

Out of 43 variables, 17 were clinical factors. These were used as input for correlation analysis with the quality outcomes. The clinical factors were further categorised as displayed in Table 1. A total of 36,116 patients were analysed in this study across two years: 2016 and 2017. Regarding the quality outcomes, there was a significant reduction in average 90-day mortality (3.56% versus 3.00%) and two-year mortality (21.38% versus 19.79%) across two years. The length of hospital stay of more than five days (70.15% versus 70.29%) and 18-month stoma rate (49.45% versus 50.93%) were insignificant. 

**Table 1:** Baseline descriptive and inferential analysis of 2016 and 2017 dataset. All variables from the 2016 and 2017 datasets were analysed. However, only relevant variables are shown in this table. ^a^Significantly different at 0.05 among its own geographical classification after post hoc adjustment. ^b^Significantly different at 0.05 between the group in 2016 and the group in 2017. ^c^Extreme outliers present in the group which is defined as 3× of the interquartile range (IQR). All the quality outcomes were risk-adjusted according to NBOCA policy. ASA: American Society of Anaesthesiologist classification; HES: hospital episode statistics database; APER: abdominal perineal resection of the rectum.

Description	Year	
	2015/2016	2016/2017
Total number of trusts in the audit (n)	146	145
Total geographical location (n)	13	19
Total number of cases reported (n)	27,757	28,661
Care pathway		
Case ascertainment (%)	95.79%	98.30%
Pre-treatment staging (%)	71.28%	77.25%^a^
Recorded performance status (%)	80.69%	88.61%
Seen by a specialist nurse (%)	94.23%^c^	94.41%^c^
Major surgery with curative intent (%)	59.00%	59.59%
Too little treatment (%)	3.97%	4.16%
Non-curative major surgery (%)	3.63%	3.48%
Too much/too frail (%)	16.65%	16.29%^a^
Not known other treatment (%)	15.55%^c^	15.68%^c^
Complexity of surgery		
ASA1 (%)	13.22%	11.51%
ASA2 (%)	52.25%	52.66%
ASA3 (%)	24.76%	26.45%
ASA4/5 (%)	2.66%^a^	2.32%
No ASA recorded (%)	3.65%^a^	3.65%
Patient with distant metastasis (%)	9.57%^b,c^	8.19%^b^
Emergency major surgery (%)	15.26%^c^	15.65%
Surgery		
Total cases of major surgery (n)	17,453	18,663
Data completeness for major surgery (%)	80.99%	82.20%
Laparoscopy attempt rate (%)	61.60%^a^	64.15%^a^
Median number of lymph nodes excised (n)	17	18
Rectal cancer		
Total number of rectal cancers having major surgery (n)	4,403	4,348
Number of rectal cancers having major surgery (n)	29.70^a^	29.21
Eighteen-month stoma number HES (n)	87^a^	82
Positive margin reported (%)	6.37%	6.57%^a^
Missing status margins (%)	24.77%	26.22%
Pre-operative radiotherapy (%)	36.42%^a^	34.82%^a^
APER (%)	24.80%	24.13%^a^
Quality outcomes		
Adjusted 90-day mortality (%)	3.56%^b^	3.00%^b,c ^
Length of hospital stay >5 days (%)	70.15%^a ^(non-adjusted)	70.29% (adjusted)
Adjusted unplanned readmission 30/90-day follow-up (%)	10.14% (90-day follow-up)	9.85% (30-day follow-up)^a^
Adjusted two-year mortality (%)	21.38%^b^	19.79%^a,b,c^
Adjusted 18-month stoma rate (%)	49.45%^a^	50.93%^a^

Correlation analysis

**Figure 2:**
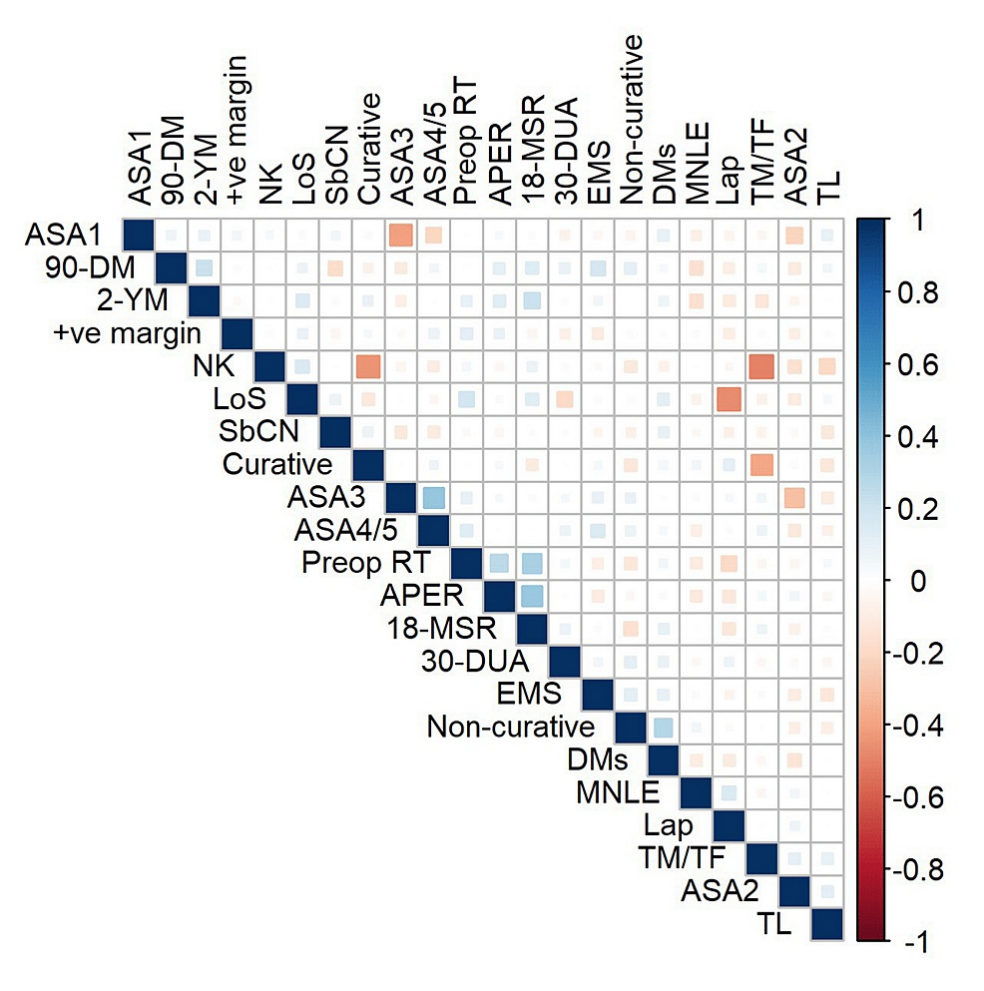
Correlation matrix diagram showing the relationship between the 17 clinical factors and five quality outcomes. Blue indicates a positive correlation, while red indicates a negative correlation. The size and colour of the dots indicate the strength of the correlation, ranging from −1 to +1. ASA: American Society of Anaesthesiologist classification; 90-DM: 90-day mortality; 2-YM: two-year mortality; +ve margin: positive margin; NK: not known; LoS: length of hospital stay >5 days; SbCN: seen by a clinical nurse; Curative: curative major resection; Preop RT: pre-operative radiotherapy; APER: abdominal-perineal excision of the rectum; 18-MSR: 18-month stoma rate; 30 DUA: 30-day unplanned readmission rate; EMS: emergency major surgery; Non-curative: non-curative; DMs: distant metastasis; MNLE: median number of lymph node excised; Lap: laparoscopic rate; TM/TF: too much/too frail; TL: too little.

Ten distinct clinical factors were found to have a significant (p < 0.05) correlation with four quality outcomes, which were the length of hospital stay of more than five days, 90-day mortality, 18-month stoma rate, and two-year mortality (Figure 2) (Table 2). The 30-day unplanned readmission rate was not significantly correlated with any clinical factors. Two-year mortality was also found to be significantly correlated (p < 0.001) with the 18-month stoma rate. 

**Table 2:** Table showing the results of significantly correlated clinical factors. *Significance level of p < 0.05. **Significance level of p < 0.01. APER: abdominal-perineal excision of the rectum.

Ninety-day mortality																		
					Patients with ASA2					Seen by a clinical nurse		Non-curative major resection				Emergency major surgery		
Ninety-day mortality	Coefficient				−0.137*					−0.154*		0.12*				0.174**		
	p-value				0.022					0.010		0.047				0.000		
Two-year mortality																		
										APER								
Two-year mortality			Coefficient							0.125*								
			p-value							0.04								
Eighteen-month stoma rate																		
					Patient with distant metastasis			Median lymph nodes excised		Laparoscopic rate				APER			Pre-operative radiotherapy	
Eighteen-month stoma rate		Coefficient			0.127*			−0.158**		−0.141*				0.390**			0.355**	
		p-value			0.162			0.007		0.017				0.000			0.000	
Length of hospital stay >5 days																		
						Emergency major surgery			Median lymph nodes excised		Laparoscopic rate				Pre-operative radiotherapy			Not known treatment pathway
Length of hospital stay >5 days				Coefficient		−0.179*			−0.169*		−0.527**				0.252**			0.207*
				p-value		0.037			0.049		0.000				0.004			0.016
Correlation between quality outcomes																		
													Eighteen-month stoma rate					
Two-year mortality							Coefficient						0.194**					
							p-value						0.001					

Regression model 

**Figure 3:**
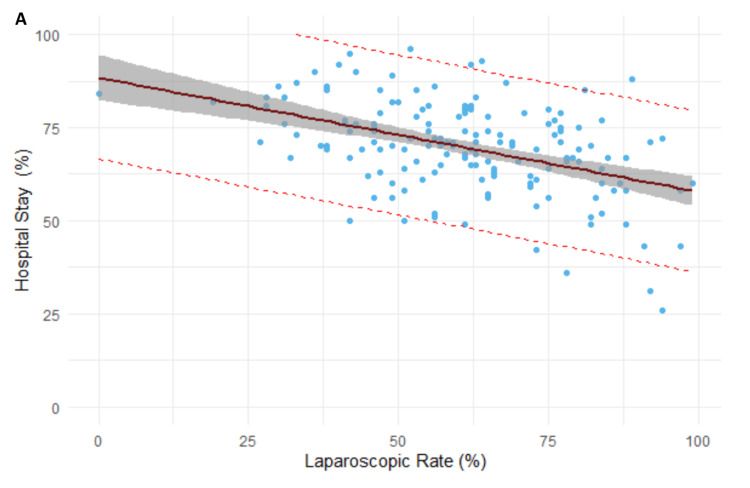
Regression model between laparoscopic rate and the length of hospital stay of greater than five days.

Four clinical factors were found to demonstrate significance with the two quality outcomes. The rate of laparoscopy was a significant predictor of the length of hospital stay of greater than five days (beta = 0.3, R^2^ = 0.317, p < 0.001). Every 1% increase in laparoscopic rates results in a reduction of 0.3% of patients remaining in hospitals postoperatively for more than five days (Figure 3). 

**Figure 4:**
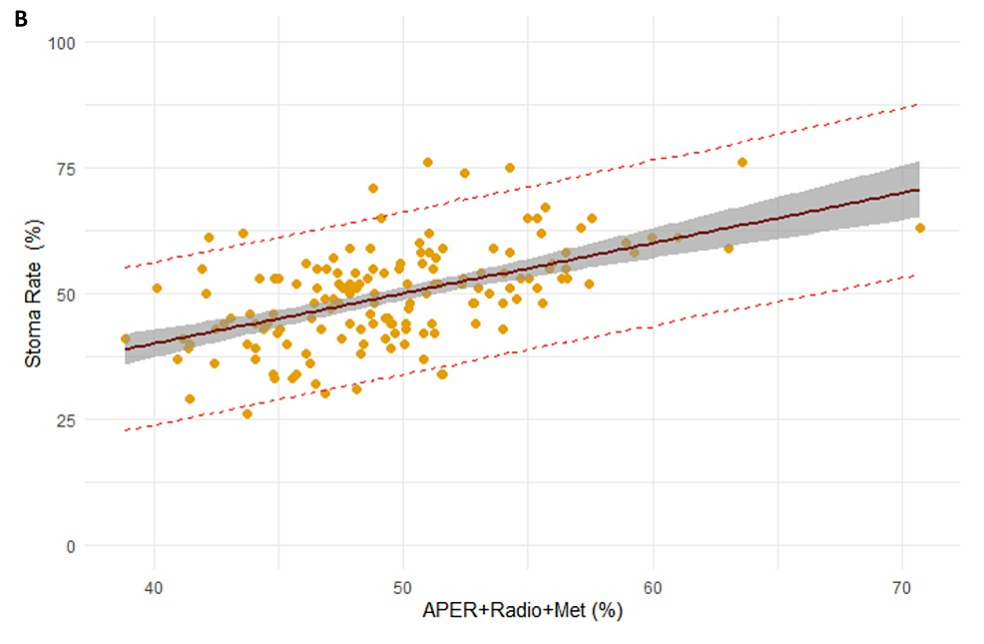
Regression model between APER rates, pre-operative radiotherapy and patients with distant metastasis and stoma formation at 18 months. APER: abdominal-perineal excision of the rectum; Radio: pre-operative radiotherapy; Met: patients with distant metastasis.

Pre-operative radiotherapy, APER rates and patients with distant metastasis were significant predictors for stoma formation at 18 months (beta = 0.6, R^2^ = 0.22, p < 0.001). Every 1% increase in the rate of all three factors led to a 0.6% increase in the rate of stoma formation (Figure 4). 

**Figure 5:**
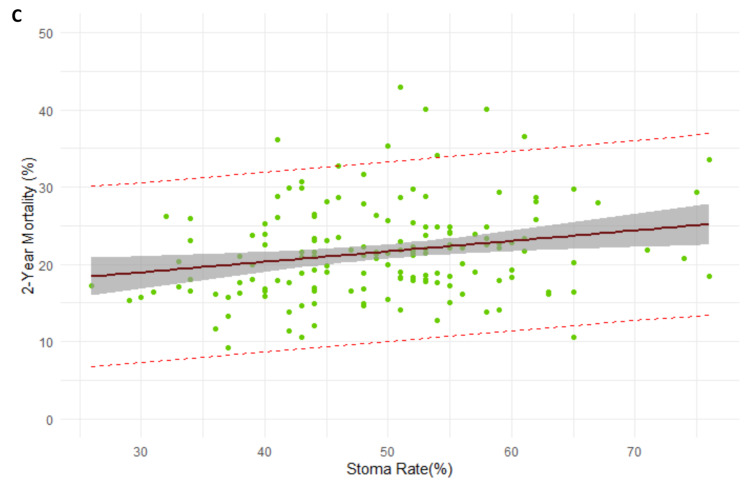
Regression model between 18-month stoma rate and two-year mortality rate.

Two quality outcomes demonstrated a significant difference between each other. The 18-month stoma rate was demonstrated to be a significant predictor of the two-year mortality rate (beta = 0.13, R^2^ = 0.042, p < 0.001). Every 1% increase in the 18-month stoma rate led to a 0.13% increase in two-year mortality (Figure 5). No clinical factors were found to have significant regression (p = 0.07) with 90-day mortality.

Validation 

The in-sample evaluation demonstrated an RMSE of 10.39, 8.81, and 6.24 for the length of hospital stay of greater than five days, the 18-month stoma rate, and the two-year mortality rate model, respectively. External validation was performed using the 2013 dataset. The length of hospitalisation could not be validated with the 2013 dataset because it was not risk-adjusted. The validated RMSE was 0.811 and 4.62 for the stoma rate and two-year mortality, respectively.

## Discussion

Our retrospective analysis of the two-year dataset for all hospitals in the NBOCA identified 10 highly correlated factors. Of those, four clinical factors (laparoscopy rate, APER, pre-operative radiotherapy, and patients with distant metastases) had a significant impact on the quality of care provided for colorectal cancer patients undergoing surgeries among the included hospitals. Interestingly, no parameters were predictors of 90-day mortality or 30-day unplanned readmission rate. Of the quality outcomes studied, we also identified that the 18-month stoma rate and two-year mortality had a strong association.

Laparoscopic surgery has been shown to shorten the duration of hospitalisation compared to laparotomy, as minimal incisions in laparoscopy accelerate the patients’ post-operative recovery [5]. It is noteworthy that the hospital laparoscopy rate in this study included both successful and unsuccessful laparoscopy attempts. A subgroup analysis of only successful laparoscopies may increase this significance further. Moreover, this study demonstrated a neutral effect on the risks of postoperative mortality and readmission rate of laparoscopy. Therefore, hospitals should consider increasing the use of laparoscopy as this is directly proportional to reducing hospitalisation beyond five days.

Aside from APER, this study discerned two additional factors that were related to the chances of stoma formation. These were pre-operative radiotherapy and patients with distant metastasis. Distant metastasis and patients requiring neoadjuvant radiotherapy have been previously proven to be risk factors for permanent stoma formation [6]. This association could be due to enhanced operative challenge and perioperative complications in patients who required neoadjuvant radiotherapy or those with advanced malignancy [6,7]. Alternatively, stoma formation at 18 months could also be the surgeons’ “temporary” measure to reduce the incidence of anastomotic leakage [8,9]. Codd et al. had previously reported that the high permanent stoma rate at his unit was due to the unit offering services such as multi-visceral surgery and that it also received many tertiary referrals [10]. Therefore, considering these factors would enable better representation of the 18-month stoma rate and, hence, the surgical quality provided at each unit.

Long-term stoma affects patients' quality of life [11], and worsens two-year survival as demonstrated by the third regression model. There is, however, a lack of studies elucidating why permanent stomas are related to mortality. Harris et al. reasoned that patients receiving permanent stomas were more likely to be frailer [12], while Lee et al. demonstrated a significant reduction in survival of 17% over five years in patients with permanent stomas compared to those without [13]. Stoma formation has wide-reaching clinical and social implications and thus should be discussed thoroughly with patients.

Limitations

One of the limitations in this study is the metric used to audit the quality of care of colorectal cancer patients in the NBOCA. The NBOCA has been evolving since it first started in 2004, which makes it difficult to compare the results between different years. Additionally, we acknowledge that the dataset was limited in 2016/2017. Nonetheless, validation of these models demonstrated that these relationships were likely to exist across 2013 and 2016-2017. The findings from this study could be used to compare with the current level of clinical practice. 

In contrast with the study by Tekkis et al., which investigated the effect of clinical factors on mortality in colorectal cancer patients using logistic regression [14], this study explored other areas of clinical outcomes. Where those significantly correlated factors did not demonstrate significant regression and achieve linearity in this study, alternative models could be trialled in the future to determine their relationship.

## Conclusions

Four clinical factors obtained from NBOCA were identified to be most significantly associated with the quality of care among hospitals. The four factors were the rate of laparoscopy, pre-operative radiotherapy, APER rates, and patients with distant metastasis. Our study showed that an increase in the rate of laparoscopy was correlated with shorter hospital stays and an increase in pre-operative radiotherapy, APER rates, and patients with distant metastasis was correlated with increased stoma formation. Hence, NBOCA should consider adjusting these factors to prevent overlooking the quality of care provided among hospitals. Clinicians should carefully consider these factors upon interpreting the results in the NBOCA, and consider these factors along with the individual patient's history when formulating a management plan for patients with colorectal cancer.
